# Peripheral blood immune markers associated with negative symptoms in first episode psychosis: a systematic review

**DOI:** 10.3389/fpsyt.2026.1782134

**Published:** 2026-04-10

**Authors:** Germaine Ingabire, Luis Felipe Scarabelot, Cristina Marta Del-Ben, Fabiana Corsi-Zuelli

**Affiliations:** 1 Department of Neuroscience and Behaviour, Ribeirão Preto Medical School, University of São Paulo, Ribeirão Preto, Brazil; 2 Department of Psychiatry, Warneford Hospital, University of Oxford, Headington, Oxford, United Kingdom

**Keywords:** cytokines, first episode psychosis (FEP), immune markers, interleukin-1β, interleukin-6, negative symptoms, peripheral blood mononuclear cells (PBMCs), tumour necrosis factor-α

## Abstract

**Background:**

Negative symptoms are a core feature of psychotic disorders, evident from the first episode of psychosis (FEP), and are strongly associated with poor functional outcomes. Increasing evidence suggests that immune dysregulation may contribute to negative symptom expression in FEP individuals; however, findings remain heterogeneous. This systematic review aimed to synthesise recent evidence on the association between peripheral blood immune markers and negative symptoms in FEP.

**Methods:**

A systematic search of PubMed and Scopus was conducted following PRISMA guidelines. Observational studies published within the last five years were included if they assessed peripheral blood immune markers and negative symptoms in individuals with FEP and included a healthy control group. Study quality was evaluated using the Newcastle-Ottawa Scale. Findings were synthesised narratively due to methodological heterogeneity.

**Results:**

Seven case-control studies met the inclusion criteria, comprising predominantly young adults with FEP, including both antipsychotic-naïve and minimally treated individuals. Across studies, a broad range of immune markers was assessed, most commonly IL-6, IL-1β, and TNF-α. FEP individuals generally showed elevated levels of inflammatory markers compared with controls. Associations between immune markers and negative symptoms were modest but consistently positive, with IL-1β and TNF-α showing the most reproducible associations with negative symptom severity. Findings for other cytokines were more variable. Notably, most studies relied on total negative symptom scores, and none examined symptom subdomains or dimensional constructs.

**Conclusions:**

Peripheral immune dysregulation, particularly involving IL-1β and TNF-α, appears to be associated with negative symptom severity in FEP. However, heterogeneity in immune measurement and symptom assessment limits mechanistic interpretation. Future studies should prioritise dimensional assessment of negative symptoms, stratification by inflammatory profiles, and integration of cellular immune phenotyping to better elucidate immune-symptom relationships in early psychosis.

## 1 Introduction

Schizophrenia and other psychotic disorders (hereafter psychosis) are severe psychiatric disorders characterized by positive symptoms (delusions and hallucinations), negative symptoms (e.g., social withdrawal, reduced motivation and pleasure, diminished expressivity), and cognitive deficits that together substantially reduce the quality of life (1–3). Antipsychotics used to treat psychotic symptoms were discovered over 70 years ago and are generally effective in alleviating positive symptoms (4, 5). However, approximately one-third of individuals with psychosis do not respond to conventional treatment with antipsychotics (6, 7), and negative symptoms remain a major unmet clinical need (8).

Negative symptoms are a fundamental aspect of psychosis and are evident even during the first episode of psychosis (FEP), significantly impacting long-term functioning (9–11). Evidence suggests that immune dysfunction, as measured by peripheral blood cytokines, is implicated in sickness behaviour, motivational withdrawal, and altered reward processing in both animal and human studies (12). Identifying immune-related markers associated with symptom domains that are difficult to treat, such as negative symptoms, will be important to guide earlier and more targeted interventions for this difficult-to-treat symptom dimension, particularly during the early stages of psychosis, with the potential to improve long-term functional outcomes.

Increasing evidence suggests that immune dysfunction is involved in the pathophysiology of some individuals with psychosis (13). Meta-analyses (14, 15) show mildly raised levels of the acute phase protein C-reactive protein (CRP) and cytokines, such as interleukin (IL)-6, IL-17, tumour necrosis factor (TNF)-α, interferon (IFN)- γ, and transforming growth factor (TGF)-β, in the blood of individuals with FEP relative to controls, even before antipsychotic treatment. Evidence of potential causality is supported by longitudinal studies showing that peripheral blood inflammation precedes the onset of psychosis (16) and by genome-wide associations studies showing a strong association between schizophrenia and the major histocompatibility complex on chromosome six, which is implicated in several immune pathways (17).

Recent studies also suggest that chronic low-grade inflammation in psychosis may contribute to the development and persistence of negative symptoms, particularly in the early stages of psychosis (18), but findings are heterogeneous across the studies. For example, the latest systematic review in FEP identified ten studies assessing associations between negative symptoms and peripheral blood immune markers (18). Levels of IL-1β, IL-6, IL-2, TNF-α, and IL-4 were positively associated with the severity of negative symptoms in FEP. Levels of IL-10 were negatively related, while no differences were found for IFN-γ, IL-8, IL-12, or IL-17. Notably, these associations were not replicated across the studies (the only exception being the IL-10 association observed in two studies). These mixed results underscore the need for further investigations into the relationship between peripheral blood immune markers and negative symptoms in FEP.

The aim of this systematic review was to provide updated evidence on the association between peripheral immune markers and negative symptoms in FEP individuals. We also provide evidence of peripheral blood immune markers in FEP compared with controls. We focused on FEP individuals to minimize confounding related to illness chronicity and long-term pharmacological treatment exposure. We did not restrict to antipsychotic-naïve samples to maximise study identification and capture a broader representation of the early-stage psychosis population.

## 2 Methods

This systematic review was conducted and reported according to the Preferred Reporting Items for Systematic Reviews and Meta-analyses (PRISMA) guidelines (19) (**Supplementary Table S1**).

### 2.1 Search strategy and study selection criteria

A systematic search was performed on December 16, 2025, using the PubMed and Scopus databases. Search terms were constructed using keywords and Medical Subject Headings (MeSH), with truncation (*) applied where appropriate to capture variant word endings. In addition to electronic searches, we manually searched relevant lists of the retrieved articles for eligible studies that may have been missed. Titles and abstracts were screened rigorously to identify potentially relevant studies. Systematic reviews and meta-analyses were excluded at the search stage.

We provided the complete search strategy in the **Supplementary Material**.

We focused on observational studies (case-control, cross-sectional, or longitudinal). Interventional studies were excluded. Articles were not restricted exclusively to antipsychotic-naïve samples in order to maximise study identification and reflect real-world early psychosis cohorts.

#### 2.1.1 Inclusion criteria

Studies were included if they met the following criteria: i) included participants with an established diagnosis of FEP; ii) measured of inflammatory markers in blood (serum or plasma); iii) evaluated negative symptoms in FEP; iv) included controls without psychosis diagnosis; v) original studies with humans; vi) written in English; vi) published within the five years preceding the final search (December 16, 2025).

#### 2.1.2 Exclusion criteria

Studies were excluded if they met one of the following criteria: i) experimental studies (e.g., *in vitro* studies, genetic studies, animal models); ii) review articles, posters, conference abstracts, editorials, letters, protocols, systematic reviews, meta-analyses, or intervention studies; iii) no measurement of peripheral blood immune markers in FEP; iv) no assessment of negative symptoms in FEP; v) not aimed to correlate/associate negative symptoms with blood inflammatory markers in FEP; vi) full text not available; vii) not written in English.

### 2.2 Data extraction and analysis

Screening and data extraction were conducted by the first author with close supervision and discussion with the fourth author. Both the first and fourth authors manually searched relevant lists of the retrieved articles for eligible studies that may have been missed. Any uncertainties were discussed until a consensus was reached. All the records were exported to Rayyan (20).

**Figure 1:**
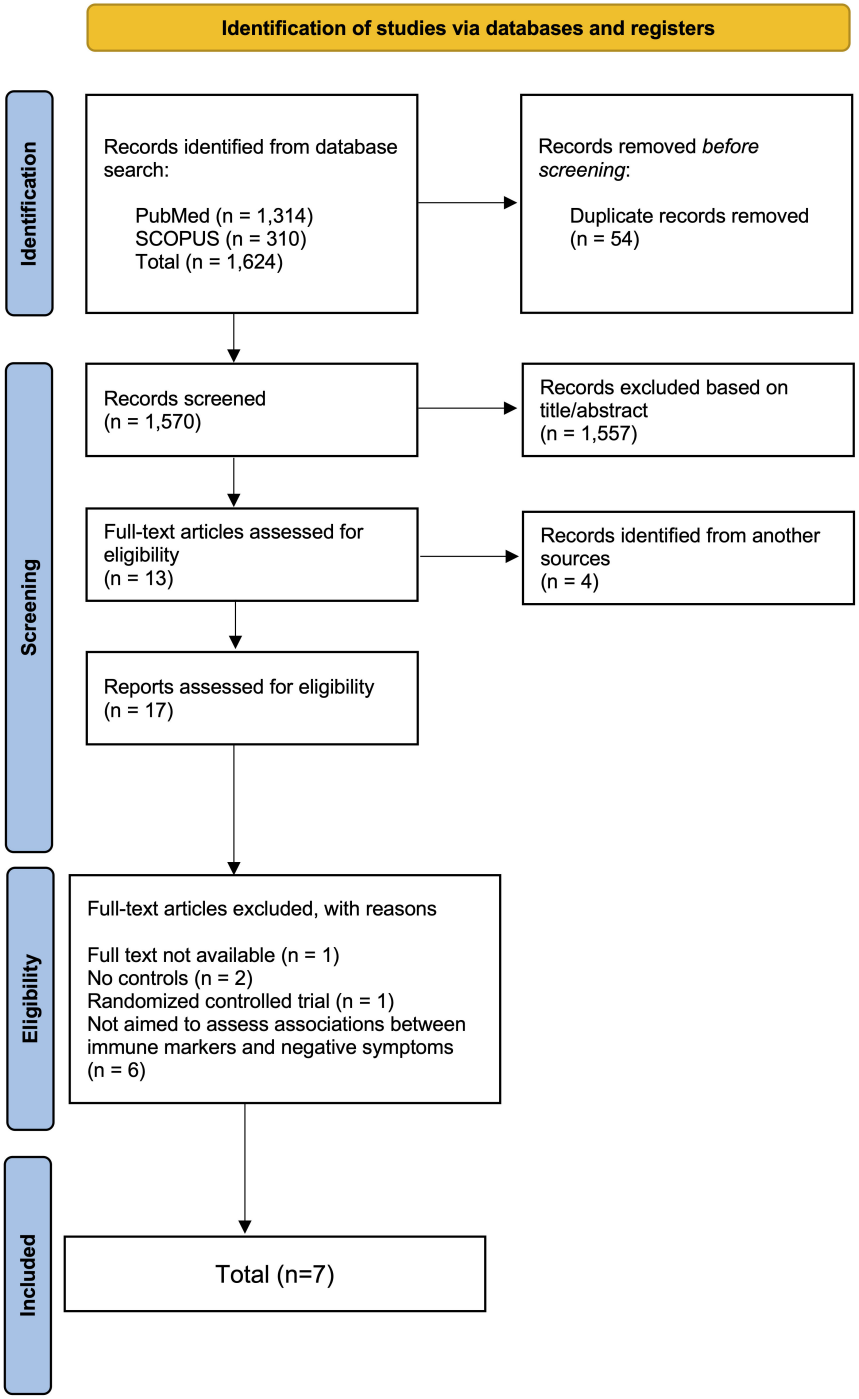
PRISMA flow diagram.

Study screening and selection were conducted using a two-stage process, consisting of title and abstract screening, followed by full-text review of potentially eligible articles. A total of 1,624 records were identified, and from these, fifty-four duplicated records were excluded. Afterwards, titles and abstracts of the remaining 1,570 articles were reviewed according to the inclusion and exclusion criteria. A total of 1,557 records were not eligible and were excluded. Thirteen articles were eligible for full-text assessment, and an additional four articles were identified through reference list screening. Of the seventeen records eligible for full-text assessment, ten studies were excluded, resulting in a total of seven studies that met the inclusion criteria. **Figure 1** illustrates the review process using the PRISMA flow diagram.

Full articles included in this review were analysed according to the following characteristics: study design, age, sex, country of residency, sample size, study population, the types of peripheral blood inflammatory markers measured, the instruments used to assess negative symptoms, and direction of the reported associations between negative symptoms and peripheral blood inflammatory markers in FEP.

### 2.3 Quality assessment and risk of bias

Quality assessments for each of the included studies were done using the Newcastle-Ottawa Scale (NOS) (21) for case-control studies (**Supplementary Table S2**). The NOS comprises eight items and evaluates study quality based on three domains: selection (representativeness and source of sample), comparability (study design and analyses), and exposure (for cohort studies). Studies were classified based on their total star rating, ranging from 0 (“poor”) to 9 (“good”).

## 3 Results

### 3.1 Overview of studies included in the systematic review

An overview of the studies included in the review is presented in **Table 1**. Seven case-control studies were included in the systematic review (22–28). Studies were conducted in China (n = 3), Serbia (n = 1), Egypt (n = 1), Brazil (n = 1), and the United States (n = 1). All studies employed cross-sectional analyses of case-control studies, comparing individuals with FEP to control participants without psychosis.

**Table 1:** Characteristics of studies included in the review.

Author (year)	Country	Study design	FEP			Controls	
			Characteristic (diagnosis)	N (male/female)	Age (mean ± SD)	N (male/female)	Age (mean ± SD)
Binic et al. (2025) (22)	Serbia	Case–control (cross-sectional)	FEP/drug naïve (ICD-10, MINI)	38 (23/15)	27.63 ± 6.84	22 (14/8)	29.91 ± 6.46
Ella et al. (2024) (23)	Egypt	Case–control (cross-sectional)	First episode schizophrenia/drug naïve (DSM IV/SCID-1)	20 (9/11)	30 ± 8.19	20 (10/10)	31.25 ± 7.37
Corsi-Zuelli et al. (2024) (28)	Brazil	Case-control (cross-sectional)	FEP/treated (DSM-IV, SCID-CV)	134 (89/45)	29.9 ± 11.9	235 (126/109)	31.6 ± 11.4
Hughes et al. (2022) (24)	USA	Case-control (cross-sectional)	FEP with affective disorder (bipolar or major depression)/treated (DSM-IV, SCID-1)	22 (16/6)	22.0 (13.2; 29.6)*	45 28/17	20.7 (13.7; 37.0)*
			FEP with non-affective disorder/treated (DSM-IV, SCID-1)	31 (26/5)	21.1 (13.3; 26.8)*		
Lin et al. (2021) (25)	China	Case-control (cross-sectional)	First episode schizophrenia/drug naïve (DSM V/SCID-1)	51 (32/19)	27.51 ± 8.71	114 (21/93)	44.31 ± 14.40
Dai et al. (2020) (26)	China	Case–control (cross-sectional)	First episode schizophrenia/drug naïve (DSM-IV)	37 (20/17)	24.2 ± 6.5	60 (32/28)	24.6 ± 3.8
Zhu et al. (2020) (27)	China	Case–control (cross-sectional)	First episode schizophrenia/drug naïve (DSM-IV, SCID-1)	119 (76/43)	29.07 ± 7.71	135 (80/55)	29.38 ± 7.21

*Expressed as minimum-maximum.

FEP, First episode psychosis; ICD-10, International Classification of Diseases, 10th Revision; SCID, Structured Clinical Interview for DSM Disorders; MINI, Mini-International Neuropsychiatric Interview.

Five studies included drug-naïve individuals with first-episode schizophrenia (22, 23, 25–27), whereas two studies included minimally treated FEP samples (24, 28). Sample sizes in FEP groups ranged from n=20 (23) to n=134 participants (28), and control groups ranged from n=20 (23) to n=235 individuals (28). Across studies, participants were predominantly young adults, with mean ages ranging from the mid-20s to early 30s, and males comprised approximately 50–65% of FEP samples in most cohorts.

Negative symptoms were assessed using the PANSS negative subscale (PANSS-N) in five studies (22, 23, 25–27), the Scale for the Assessment of Negative Symptoms (SANS) in one study (24), and an OPCRIT-derived negative symptom factor in another study (28). Higher PANSS-N, SANS, and OPCRIT-derived negative symptom factor scores indicate higher levels of negative symptoms. Mean PANSS-N scores across studies ranged from approximately 19 to 30, indicating moderate negative symptom severity (**Table 2**).

**Table 2:** Peripheral immune markers and negative symptoms in FEP individuals.

Author (year)	Negative symptom (mean ± SD)^a^	Peripheral blood immune marker	Association between blood immune markers and negative symptom severity^b^	Total NOS (out of 9)
Binic et al. (2025) (22)	PANSS-N (23.45 ± 6.34)	IL-6, IL-1β, IL-10, IL-2	IL-1β positive correlation (ρ = 0.374, p = 0.021)	8
Ella et al. (2024) (23)	PANSS-N (30.35 ± 5.34)	IL-6	IL-6 NS (r = −0.228, p = 0.333)	8
Corsi-Zuelli et al. (2024) (28)	OPCRIT (bifactor model)	IL-6, IL-1β, IL-10, TNF-α, TGF-β, IFN-γ, IL-4	IL-1β positive association (beta = 0.25, p=0.013)	8
Hughes et al. (2022)^c^ (24)	SANS: 7 (3-8)^d^	IL-6, IL-1β, IL-10, IL-2, TNF-α, TGF-β, IFN-γ, IL-4, IL1-α, IL-15, TNF-β, IFN-α2, Eotaxin, IL-8, CXCL10/IP-10, CCL2/MCP-1, CCL4/MIP-1β, IL-12 (p40), IL-12 (p70), IL-5 IL-13, IL-17, G-CSF GM-CSF, IL-7	IL-1β: positive correlation (ρ=0.334., p=0.02) IL-6: positive correlation (ρ=0.346., p=0.016) TNF-α: positive correlation (ρ=0.314., p=0.03) IFN-γ: positive correlation (ρ=0.390., p=0.006) IL-17: positive correlation(ρ=0.347, p=0.016) IL-4: positive correlation (ρ=0.354., p=0.014) Eotaxin positive correlation (ρ=0.353, p=0.014) GM-CSF positive correlation (ρ=0.366, p=0.011) IL-12(p40): positive correlation (ρ=0.287, p=0.048)	6
Lin et al. (2021) (25)	PANSS-N (18.94 ± 7.49)	TNF-α	TNF-α NS (beta = 0.14, p = 0.360)	6
Dai et al. (2020) (26)	PANSS-N (24.1 ± 3.3)	IL-6, IL-1β, S100β, NGF, NT-3	IL-1β positive association (beta = 0.811, p<0.001)	7
Zhu et al. (2020) (27)	PANSS-N (26.89 ± 7.33)	TNF-α, SOD, GSH-Px, CAT, MDA	TNF-α positive association (r = 0.37, p<0.001)	8

a Higher scores on PANSS-N, SANS and OPCRIT indicate higher levels of negative symptoms.

b Positive associations/correlations indicate that higher immune marker levels were related to greater negative symptom severity.

c Immune markers measured from supernatant from PBMCs (media only).

d Results expressed as median (interquartile range).

PANSS-N, Positive and Negative Syndrome Scale-Negative Subscale; OPCRIT, Operational Criteria Checklist for Psychotic Illness; SANS, Scale for the Assessment of Negative Symptoms; NS, Non-significant; ρ, Spearman’s correlation coefficient; r, Pearson’s correlation coefficient; IL, Interleukin; TNF, Tumor Necrosis Factor; TGF, Transforming Growth Factor; IFN, Interferon; CXCL, Motif Chemokine Ligand; CCL, Motif Chemokine Ligand; G-CSF, Granulocyte Colony-Stimulating Factor; GM-CSF, Granulocyte-Macrophage Colony-Stimulating Factor; S100β, Calcium-Binding Protein Beta; NGF, Nerve Growth Factor; NT3, Neurotrophin-3; SOD, Superoxide Dismutase; GSH, Glutathione; CAT, Catalase; MDA, Malondialdehyde NOS, Newcastle–Ottawa Scale.

Cytokines were measured through blood sampling with prior fasting (24–27) or with unspecified protocols (22, 23, 28). Confounding factors were inconsistently controlled in the analyses across the included studies. Detailed information about confounding factors is provided in **Supplementary Table S2**.

### 3.2 Methodological quality

Methodological quality, assessed using the NOS, ranged from 6 to 8 out of 9 points, indicating overall moderate to good quality across studies (**Table 2**). In general, all studies clearly defined cases using standardized diagnostic criteria (DSM-IV, DSM-V, or ICD-10) and structured diagnostic interviews (SCID or MINI), and control groups were consistently defined as psychiatrically healthy individuals.

Limitations were primarily related to incomplete control for potential confounders (e.g., body mass index or smoking status in some studies) and, in one study, an imbalance in sex distribution between cases and controls (25). Nevertheless, most studies achieved high NOS scores due to robust case definitions and exposure ascertainment.

### 3.3 Peripheral blood immune markers in FEP compared with controls

A wide range of peripheral immune markers were assessed across studies (**Table 2**; **Supplementary Table S3**). Six studies measured immune-related markers in the peripheral blood (serum or plasma), with only one exception (24), which measured immune-related markers from the supernatant of unstimulated (media only) or stimulated (e.g., lipopolysaccharide, LPS) peripheral blood mononuclear cells (PBMCs).

The most frequently measured cytokines were IL-6 (five studies), IL-1β (four studies), and TNF-α (four studies). IL-10 was measured in three studies, while IL-2, TGF-β, IFN-γ, and IL-4 were measured in two studies. Other immune-related markers (IL1-α, IL-15, TNF-β, IFN-α2, Eotaxin, IL-8, CXCL10/IP-10, CCL2/MCP-1, CCL4/MIP-1β, IL-12 (p40), IL-12 (p70), IL-5, IL-13, IL-17, G-CSF, GM-CSF, IL-7, S100β, NGF, NT-3) were measured by a single study (24), while another (27) also measured oxidative stress markers concomitantly to cytokines in the peripheral blood of participants (SOD, GSH-Px, CAT, MDA).

Overall, despite the methodological heterogeneity across the studies, FEP individuals exhibited higher levels of several inflammatory cytokines and other immune-related markers compared with controls (**Supplementary Table S3**). For example, IL-6 was elevated in FEP relative to controls in four of the five studies that assessed this cytokine (23, 26–28), with one study reporting no significant differences (22). IL-1β concentrations were significantly higher in FEP participants relative to controls in three of four studies (22, 26, 28). One study reported no between-group differences on IL-1β, but with a different methodology (measures from PBMCs supernatants) (24). TNF-α levels were consistently elevated in FEP relative to controls in all four studies that measured this cytokine (24, 25, 27, 28).

Out of the three studies that measured IL-10, two found significantly elevated levels in FEP versus controls (22, 28). For other cytokines (e.g., IL-2, TGF-β, IFN-γ, and IL-4), these were measured in two studies with different methodologies, and findings were inconsistent, with elevated levels not replicated across the studies.

Oxidative stress markers measured in peripheral blood showed a mixed pattern, with reduced GSH-Px and elevated MDA in FEP, while SOD and CAT did not differ from controls (27).

Several cytokines measured from unstimulated PBMC supernatant were significantly higher in FEP individuals with affective psychosis relative to controls, in particular IL-1β, IL-6, TNF-α, IFN-γ, IL-4, and IL-17 (24).

### 3.4 Association between peripheral blood immune markers and negative symptoms in FEP

Associations between negative symptoms and peripheral blood immune markers are presented in **Table 2**.

Overall, associations between immune-related markers and negative symptoms were of moderate strength and positively related, suggesting higher levels of immune markers associated with more severe negative symptoms, with no study reporting inverse associations.

IL-1β showed the most consistent association with negative symptoms. Significant positive associations between IL-1β levels and negative symptom severity were reported in all four studies that assessed this relationship (22, 24, 26, 28). Correlation coefficients and regression estimates indicated moderate effect sizes, suggesting that higher IL-1β concentrations were associated with more severe negative symptoms.

From the four studies that assessed associations between TNF-α levels and negative symptoms, significant positive associations indicating higher TNF-α and worse negative symptoms were found in two studies (24, 27). No significant association was found in the other two studies (25, 28).

Findings for IL-6 were mixed. Of the five studies assessing associations with negative symptoms, only the study (24) reported a significant positive association between higher IL-6 levels measured in unstimulated PBMC supernatants and more severe negative symptoms measured by SANS. This association was observed specifically in FEP individuals with affective psychosis, but not in non-affective FEP individuals.

The study with unstimulated PBMCs (24) also found significant positive associations between higher levels of other immune markers (specifically IFN-γ, IL-4, IL-17, eotaxin, GM-CSF, and IL-12p40) and more severe negative symptoms measured by SANS total scores. The IFN-γ and IL-4 associations were not replicated in another study that measured these markers in plasma and evaluated negative symptoms using the OPCRIT (28). Eotaxin, GM-CSF, and IL-12 (p40) were not assessed by other studies.

## 4 Discussion

In this systematic review, we synthesised evidence published within the last five years examining the relationship between peripheral blood immune markers and negative symptoms in FEP. Overall, the findings suggest that pro-inflammatory immune activity is present early in the course of psychosis and that specific cytokines – most consistently IL-1β and TNF-α – may be associated with greater negative symptom severity. Collectively, the findings point to a possible relationship between heightened immune activity and greater negative symptom burden in early psychosis, while highlighting the need for more refined approaches to both negative symptom assessment and immune phenotyping in FEP.

The findings of this review are broadly consistent with and extend previous work (18) evaluating the relationship between peripheral blood immune markers and negative symptoms in FEP. In line with Dunleavy et al. (2022), we observed evidence of elevated peripheral blood inflammation in FEP, including among antipsychotic-naïve individuals, alongside modest but reproducible associations between inflammation and negative symptoms, most notably IL-1β and TNF-α. Cytokines such as IL-1β and TNF-α are central mediators of immune activation and have been widely implicated in sickness behaviour, motivational withdrawal, and altered reward processing in both animal and human models (12). Their association with negative symptoms across studies supports the hypothesis that low-grade inflammatory processes may contribute to the emergence or persistence of these symptoms early in psychosis.

Regarding IL-6, previous work using structural equation modelling reported significant positive associations between circulating IL-6 levels and negative symptoms across early and chronic stages of psychosis (29). Findings in FEP included in the present systematic review were less consistent. This variability may reflect both methodological differences and the complex biology of IL-6. From a mechanistic perspective, IL-6 is a pleiotropic cytokine that can exert pro-inflammatory effects via *trans-signalling* pathways (involving soluble IL-6 receptor, IL-6R) or anti-inflammatory and regenerative effects via *classical signalling* through the membrane-bound receptor (30). These findings suggest that simply measuring total IL-6 concentrations may be insufficient to capture its functional relevance. Future studies should therefore consider assessing IL-6 pathway activity more comprehensively, for example, by measuring soluble IL-6R and glycoprotein 130 alongside IL-6 itself, as has been done in depression research to better index biologically active IL-6 signalling (30).

Beyond cytokines measured in plasma/serum, we extend the previous literature and show evidence of associations between negative symptoms and immune markers assessed from PBMC supernatants. In particular, the study by Hughes et al. (2022) reported associations between negative symptoms and IFN-γ, IL-4, IL-17, eotaxin, GM-CSF, and IL-12p40 (24). These immune markers span multiple immune pathways, including CD4+ T cells (e.g., Th1, Th2, Th17) and myeloid-related responses, suggesting that immune dysregulation associated with negative symptoms may not be restricted to a single inflammatory axis. Importantly, these findings indicate that functional immune assays capturing cytokine production capacity may reveal associations that are not detectable when measuring circulating cytokine levels alone.

However, measuring total PBMC-derived cytokine output does not provide information about the specific cellular sources driving immune alterations. PBMCs comprise a heterogeneous mixture of immune cell populations, including CD4^+^ T helper cells (Th1, Th2, Th17), regulatory T cells (Tregs), CD8^+^ T cells, B cells, natural killer cells, and monocytes. Dissecting immune dysregulation at the cellular level may therefore be critical for advancing mechanistic understanding. Emerging evidence suggests that a hypofunctional state of Tregs – key modulators of immune homeostasis in the periphery and in the brain – may contribute to the disinhibited, low-grade inflammatory state observed in psychosis (31). Some studies have further reported associations between reduced Treg number or function and greater negative symptom severity (32). Extending these findings to larger and well-characterised FEP samples using deep immune phenotyping and functional assays will be essential to clarify cellular mechanisms linking immune dysfunction to negative symptoms.

A key limitation across the reviewed studies concerns how negative symptoms were conceptualised and measured. Most studies relied on total negative symptom scores, primarily derived using the PANSS negative subscale or the SANS. Only the one study applied a more sophisticated approach, using OPCRIT data combined with item response modelling and factor analysis to derive dimensional symptom constructs (28). Still, none of the included studies formally examined negative symptoms subdomains or individual symptom components. This is a critical limitation, given accumulating evidence that negative symptoms are not a unitary construct (33, 34).

Factor analytic work (33–36) consistently supports the existence of two higher-order dimensions of negative symptoms, with further differentiation into five lower-order domains, namely, diminished emotional expression (including blunted affect and alogia) and motivation-pleasure deficits (including anhedonia, avolition, and asociality). From a biological perspective, this distinction is highly relevant. Work by Goldsmith et al. (37–41) has emphasised that motivational and reward-related negative symptoms, such as anhedonia, avolition, and asociality, may be particularly sensitive to inflammatory processes, given their overlap with neural circuits involved in effort-based decision-making and reward valuation. In contrast, expressive deficits (e.g., blunted affect) may reflect more trait-like or neurodevelopmental mechanisms. By collapsing these domains into a single score, most studies in this review may have obscured domain-specific immune associations, potentially contributing to inconsistent findings.

Supporting the dimensional framework and its relation to inflammation, a previous machine learning study of recent-onset psychosis identified a subgroup characterized by prominent anhedonia that showed significantly higher levels of IL-6, S100β, and IL1RA, alongside lower IFN-γ levels (42). This highlights the importance of examining specific negative symptom profiles rather than relying solely on total scores. Notably, within the studies included in this review, none formally separated the two high-order negative symptom domains. The study by Binic et al. (2025) explored item-level associations in addition to total negative symptoms scores, although no significant associations were observed, likely reflecting limited statistical power due to modest sample size. Together, these findings underscore a critical gap in the literature and point to the need for more granular symptom phenotyping in future studies.

Our systematic review has limitations. One important consideration is the clinical heterogeneity among FEP individuals. The included studies comprised mixed populations of first-episode schizophrenia, non-affective psychosis, and affective psychosis, with variability in antipsychotic exposure (drug-naïve *versus* minimally treated). Only one study examined diagnostic subtype differences, finding higher levels of TNF-α and IL-1β (measured from PBMC supernatants) in affective psychosis compared to non-affective psychosis, with higher levels of these cytokines also associated with greater negative symptom severity in the affective group (24). These findings would align with evidence from chronic psychosis samples, in which blood immune marker levels differed between affective and non-affective diagnoses (43). Further investigation is needed to better understand the role of diagnostic subtype, illness stage, and treatment exposure on inflammatory profiles and their associations with negative symptoms.

Another important consideration relates to the assessment of negative symptoms and potential confounding factors. Negative symptoms may be primary or secondary to other clinical features, such as depressive symptoms, positive symptom severity, or medication effects. Although the included studies focused on FEP samples that were drug-naïve or minimally treated, the potential confounding influence of co-occurring symptom domains warrants further investigation. For example, more severe cognitive and positive symptoms have also been associated with elevated blood levels of IL-6, TNF-α, and IL-1β, with low to moderate effect sizes (12, 22, 28, 44). In addition, sex differences represent a relevant but underexplored factor. Previous studies have reported sex-related differences in cytokine levels among antipsychotic-naïve patients, as well as distinct trajectories of cytokine variation following treatment (45). Due to limited data, we were unable to examine sex-specific effects in the present review, highlighting the need for future studies to address sex-stratified analyses. Finally, methodological factors such as timing of blood sample collection, smoking status, age, body mass index, inflammatory comorbidities, and acute stress exposure may substantially influence peripheral immune marker levels and should be more consistently reported and controlled for in future research.

Several implications emerge from this systematic review. First, future studies should move beyond total negative symptom scores and explicitly examine symptom subdomains or dimensions, ideally using second-generation negative symptom scales or dimensional modelling approaches. Although this will require larger samples, it is essential for achieving biological specificity. Second, stratifying patients based on inflammatory profiles (e.g., low *vs*. high inflammation) may increase the chances of detecting immune-symptom associations and help identify clinically meaningful subgroups. Third, integrating cellular immune measures, such as immune cell phenotyping and functional assays, alongside cytokine measurements, may provide a more comprehensive and mechanistically informative understanding of immune dysregulation in early psychosis.

## 5 Conclusion

In conclusion, this systematic review suggests that peripheral blood immune dysregulation – most consistently involving IL-1β and TNF-α – may be associated with negative symptom severity in FEP individuals. However, these associations are modest and highly dependent on how both immune markers and negative symptoms are measured. Negative symptoms are not a unitary construct, and immune markers are not interchangeable indicators of inflammation. Progress in this field will require more precise symptom phenotyping, harmonised immune methodologies, and stratified analytic approaches. By aligning biological measures with clinically meaningful symptom dimensions, future research may clarify which patients are most likely to experience inflammation-related negative symptoms and inform targeted therapeutic strategies.

## Data Availability

The original contributions presented in the study are included in the article/**Supplementary Material**. Further inquiries can be directed to the corresponding author.

## References

[1] Owen MJ Sawa A Mortensen PB . Schizophrenia. Lancet. (2016) 388 (10039):86–97. doi: 10.1016/S0140-6736(15)01121-6, PMID: 26777917 PMC4940219

[2] Hjorthøj C Stürup AE McGrath JJ Nordentoft M . Years of potential life lost and life expectancy in schizophrenia: a systematic review and meta-analysis. Lancet Psychiatry. (2017) 4 (4):295–301. doi: 10.1016/S2215-0366(17)30078-0, PMID: 28237639

[3] Anhoque CF Biccas-neto L Domingues SCA Domingues RB . Cognitive impairment is correlated with reduced quality of life in patients with clinically isolated syndrome. (2012) 71 (2):74–7. doi: 10.1590/s0004-282x2013005000004, PMID: 23295369

[4] Leucht S Cipriani A Spineli L Mavridis D Örey D Richter F . Comparative efficacy and tolerability of 15 antipsychotic drugs in schizophrenia: a multiple-treatments meta-analysis. (2014) 382 (9896):951–62. doi: 10.1016/s0140-6736(13)60733-3, PMID: 23810019

[5] González-Rodríguez A Monreal JA Natividad M Seeman MV . Seventy Years of Treating Delusional Disorder with Antipsychotics: A Historical Perspective. Biomedicines. (2022) 10 (12):3281. doi: 10.3390/biomedicines10123281, PMID: 36552037 PMC9775530

[6] Samara MT Nikolakopoulou A Salanti G Leucht S . How many patients with schizophrenia do not respond to antipsychotic drugs in the short term? An analysis based on individual patient data from randomized controlled trials. (2019) 45:639–46. doi: 10.1093/schbul/sby095, PMID: 29982701 PMC6483567

[7] Caspi A Davidson M Tamminga CA . Treatment-refractory schizophrenia. Dialogues Clin Neurosci. (2004) 6 (1):61–70. doi: 10.31887/DCNS.2004.6.1/acaspi, PMID: 22034144 PMC3181784

[8] Galderisi S Kaiser S Bitter I Nordentoft M Mucci A Sabé M . EPA guidance on treatment of negative symptoms in schizophrenia. Eur Psychiatry. (2021) 64 (1):e21. doi: 10.1192/j.eurpsy.2021.13, PMID: 33726883 PMC8057437

[9] Bruhn D Hwang S Howarth A Dubé S . The burden of illness for patients with schizophrenia and primary negative symptoms: a systematic literature review. Schizophr Res. (2022) 248:341–4. doi: 10.1016/j.schres.2022.09.017. PMID: 36202050

[10] Howes O Fusar-Poli P Osugo M . Treating negative symptoms of schizophrenia: current approaches and future perspectives. Br J Psychiatry. (2023) 223 (1):332–335. doi: 10.1192/bjp.2023.57, PMID: 37272623

[11] Pelizza L Maestri D Leuci E Quattrone E Azzali S Paulillo G . Negative symptom configuration in patients with first episode affective psychosis: findings from the 1-year follow-up of the "Parma Early Psychosis" program. Acta Biomed. (2021) 92 (4):e2021224. doi: 10.23750/abm.v92i4.11115, PMID: 34487088 PMC8477119

[12] de Bartolomeis A Barone A Vellucci L Mazza B Austin MC Iasevoli F . Linking inflammation, aberrant glutamate-dopamine interaction, and post-synaptic changes: translational relevance for schizophrenia and antipsychotic treatment: a systematic review. Mol Neurobiol. (2022) 59:6460–501. doi: 10.1007/s12035-022-02976-3. PMID: 35963926 PMC9463235

[13] Upthegrove R Corsi-Zuelli F Couch ACM Barnes NM Vernon AC . Current Position and Future Direction of Inflammation in Neuropsychiatric Disorders: A Review. JAMA Psychiatry. (2025) 82 (10):1030–1046. doi: 10.1001/jamapsychiatry.2025.1369, PMID: 40632530

[14] Halstead S Siskind D Amft M Wagner E Yakimov V Liu Z . Alteration patterns of peripheral concentrations of cytokines and associated inflammatory proteins in acute and chronic stages of schizophrenia: a systematic review and network meta-analysis. Lancet Psychiatry. (2023) 10:260–71. doi: 10.1016/s2215-0366(23)00025-1. PMID: 36863384

[15] Howes D . A meta-analysis of immune parameters, variability, and assessment of modal distribution in psychosis and test of the immune subgroup hypothesis. (2019) 45:1120–33. doi: 10.1093/schbul/sby160, PMID: 30407606 PMC6737479

[16] Khandaker GM Pearson RM Zammit S Lewis G Jones PB . Association of serum interleukin 6 and C-reactive protein in childhood with depression and psychosis in young adult life: a population-based longitudinal study. JAMA Psychiatry. (2014) 71 (10):1121–1128. doi: 10.1001/jamapsychiatry.2014.1332, PMID: 25133871 PMC4561502

[17] Trubetskoy V Pardiñas AF Qi T Panagiotaropoulou G Awasthi S Bigdeli TB . Mapping genomic loci implicates genes and synaptic biology in schizophrenia check for updates. Nature. (2022) 604:502–8. doi: 10.1038/s41586-022-04434-5. PMID: 35396580 PMC9392466

[18] Dunleavy C Elsworthy RJ Wood SJ Aldred S . Inflammation in first-episode psychosis: the contribution of inflammatory biomarkers to the emergence of negative symptoms, a systematic review and meta-analysis. (2022), 6–20. doi: 10.1111/acps.13416, PMID: 35202480 PMC9310618

[19] Page MJ McKenzie JE Bossuyt PM Boutron I Hoffmann TC Mulrow CD . The PRISMA 2020 statement: an updated guideline for reporting systematic reviews. BMJ. (2021) 372:n71. doi: 10.1136/bmj.n71, PMID: 33782057 PMC8005924

[20] Ouzzani M Hammady H Fedorowicz Z Elmagarmid A . Rayyan-a web and mobile app for systematic reviews. Syst Rev. (2016) 5 (1):210. doi: 10.1186/s13643-016-0384-4. PMID: 27919275 PMC5139140

[21] Gualdi-russo E . The newcastle – ottawa scale for assessing the quality of studies in systematic reviews. (2026), 1–9. doi: 10.3390/publications14010004, PMID: 41725453

[22] Binic I Petrovic J Zikic O Golubovic ST Djordjevic V Stevanovic M . Clinical relevance of peripheral interleukins in drug-naive first-episode psychosis: symptom-specific associations from the PANSS dimensions. (2025) 15 (9):1–16. doi: 10.3390/brainsci15090932, PMID: 41008291 PMC12468177

[23] Ella EIAE Rabie ES Sheikh MME Ghamry RHE Hotar MS Gabrielle FF . Assessment of serum interleukin 6 in a sample of Egyptian patients with schizophrenia. Midd East Curr Psychiatry. (2024) 31. doi: 10.1186/s43045-024-00409-6. PMID: 41863001

[24] Hughes HK Yang H Lesh TA Carter CS Ashwood P . Evidence of innate immune dysfunction in first-episode psychosis patients with accompanying mood disorder. J Neuroinflamm. (2022) 19:287. doi: 10.1186/s12974-022-02648-y. PMID: 36463221 PMC9719666

[25] Lin C Chen K Yu J Feng W Fu W Yang F . Relationship between TNF-α levels and psychiatric symptoms in first-episode drug-naïve patients with schizophrenia before and after risperidone treatment and in chronic patients. BMC Psychiatry. (2021) 21 (1):561. doi: 10.1186/s12888-021-03569-5. PMID: 34763685 PMC8588730

[26] Dai N Jie H Duan Y Xiong P Xu X Chen P . Different serum protein factor levels in first-episode drug-naive patients with schizophrenia characterized by positive and negative symptoms. Psychiatry Clin Neurosci. (2020) 74:472–9. doi: 10.1111/pcn.13078. PMID: 32478952

[27] Zhu S Zhao L Fan Y Lv Q Wu K Lang X . Interaction between TNF-α and oxidative stress status in first-episode drug-naïve schizophrenia. Psychoneuroendocrinology. (2020) 114:104595. doi: 10.1016/j.psyneuen.2020.104595. PMID: 32036201

[28] Corsi-Zuelli F Quattrone D Ragazzi TCC Marcelino Loureiro CM Shuhama R Menezes PR . Transdiagnostic dimensions of symptoms and experiences associated with immune proteins in the continuity of psychosis. Psychol Med. (2024) 54:2099–111. doi: 10.1017/s0033291724000199. PMID: 38414355

[29] Corsi-Zuelli F Donohoe G Griffiths S Del-Ben CM Watson AJ Burke T . Depressive and negative symptoms in the early and established stages of schizophrenia: integrating structural brain alterations, cognitive performance, and plasma interleukin-6 levels. Biol Psychiatry Global Open Sci. (2024), 100429. doi: 10.1016/j.bpsgos.2024.100429, PMID: 39911538 PMC11795630

[30] Schaper F Rose-john S . Cytokine & growth factor reviews interleukin-6: biology, signaling and strategies of blockade IL-6. Cytokine Growth Fact Rev. (2015) 26:475–87. doi: 10.1016/j.cytogfr.2015.07.004. PMID: 26189695

[31] Corsi-zuelli F Deakin B . Neuroscience and biobehavioral reviews impaired regulatory T cell control of astroglial overdrive and microglial pruning in schizophrenia. Neurosci Biobehav Rev. (2021) 125:637–53. doi: 10.1016/j.neubiorev.2021.03.004. PMID: 33713699

[32] Corsi-zuelli F Deakin B Haruo M Lima F De Qureshi O Barnes NM . Brain, behavior, & immunity - health T regulatory cells as a potential therapeutic target in psychosis? Current challenges and future perspectives. Brain Behav Immun Health. (2021) 17:100330. doi: 10.1016/j.bbih.2021.100330. PMID: 34661175 PMC7611834

[33] Ahmed AO Kirkpatrick B Galderisi S Mucci A Rossi A Bertolino A . Cross-cultural validation of the 5-factor structure of negative symptoms in schizophrenia. Schizophr Bull. (2019) 45:305–14. doi: 10.1093/schbul/sby050. PMID: 29912473 PMC6403061

[34] Strauss GP Hong LE Gold JM Buchanan RW McMahon RP Keller WR . Factor structure of the brief negative symptom scale. Schizophr Res. (2012) 142:96–8. doi: 10.1016/j.schres.2012.09.007. PMID: 23062750 PMC3502636

[35] Strauss GP Nuñez A Ahmed AO Barchard KA Granholm E Kirkpatrick B . The latent structure of negative symptoms in schizophrenia. JAMA Psychiatry. (2018) 75:1303. doi: 10.1001/jamapsychiatry.2018.2475. PMID: 30208377 PMC6583036

[36] Strauss GP Cohen AS . A transdiagnostic review of negative symptom phenomenology and etiology. Schizophr Bull. (2017) 43:712–29. doi: 10.1093/schbul/sbx066. PMID: 28969356 PMC5472109

[37] Goldsmith DR Haroon E Miller AH Addington J Bearden C Cadenhead K . Association of baseline inflammatory markers and the development of negative symptoms in individuals at clinical high risk for psychosis. Brain Behav Immun. (2019) 76:268–74. doi: 10.1016/j.bbi.2018.11.315. PMID: 30496778 PMC6348114

[38] Goldsmith DR Rapaport MH . Inflammation and negative symptoms of schizophrenia: implications for reward processing and motivational deficits. Front Psychiatry. (2020) 11:46. doi: 10.3389/fpsyt.2020.00046. PMID: 32153436 PMC7044128

[39] Goldsmith DR Massa N Miller BJ Miller AH Duncan E . The interaction of lipids and inflammatory markers predict negative symptom severity in patients with schizophrenia. NPJ Schizophr. (2021) 7 (1):50. doi: 10.1038/s41537-021-00179-8. PMID: 34671033 PMC8528914

[40] Goldsmith DR Haroon E Miller AH Strauss GP Buckley PF Miller BJ . TNF-α and IL-6 are associated with the deficit syndrome and negative symptoms in patients with chronic schizophrenia. Schizophr Res. (2018) 199:281–4. doi: 10.1016/j.schres.2018.02.048. PMID: 29499967 PMC6111000

[41] Goldsmith DR Ning CS Strauss GP Gross RE Cooper JA Wommack EC . Inflammation is associated with avolition and reduced resting state functional connectivity in corticostriatal reward circuitry in patients with schizophrenia. Neuropsychopharmacology. (2025) 50 (11):1706–1714. doi: 10.1038/s41386-025-02114-2, PMID: 40274974 PMC12436628

[42] Lalousis PA Malaviya A Khatibi A Saberi M Kambeitz-Ilankovic L Haas SS . Anhedonia as a potential transdiagnostic phenotype with immune-related changes in recent-onset mental health disorders. Biol Psychiatry. (2024) 96 (7):615–622. doi: 10.1016/j.biopsych.2024.05.019. PMID: 38823495

[43] Brambilla P Bellani M Isola M Bergami A Marinelli V Dusi N . Increased M1/decreased M2 signature and signs of Th1/Th2 shift in chronic patients with bipolar disorder, but not in those with schizophrenia. Transl Psychiatry. (2014) 4:e406–e406. doi: 10.1038/tp.2014.46. PMID: 24984193 PMC4119216

[44] Patlola SR Donohoe G McKernan DP . The relationship between inflammatory biomarkers and cognitive dysfunction in patients with schizophrenia: a systematic review and meta-analysis. Prog Neuro-Psychopharmacol Biol Psychiatry. (2023) 121:110668. doi: 10.1016/j.pnpbp.2022.110668. PMID: 36283512

[45] Ratke I Torsvik A Bartz-Johannessen CA Fathian F Joa I Reitan SMK . Sex differences in the peripheral levels of cytokines during 12-month antipsychotic treatment in a drug-naïve schizophrenia spectrum cohort. Brain Behav Immun Health. (2025) 44:100959. doi: 10.1016/j.bbih.2025.100959. PMID: 39990282 PMC11846924

